# Delayed Corneal Healing After the Use of Topical Ophthalmic Anesthetics

**DOI:** 10.7759/cureus.70455

**Published:** 2024-09-29

**Authors:** Darby D Miller, Isabella V Wagner, Richard D Ten Hulzen, Syril Dorairaj, Arman Mashayekhi, Chelsey Krambeer, Nithya Boopathiraj, Michael Stewart

**Affiliations:** 1 Ophthalmology, Mayo Clinic, Jacksonville, USA; 2 Opthalmology, Mayo Clinic, Jacksonville, USA

**Keywords:** case report, cornea, corneal abrasion, epithelial toxicity, ophthalmic anesthetics

## Abstract

Topical anesthetics for control of pain from corneal abrasions can cause drug-related toxicity that delays corneal healing. This report documents three cases where topical anesthetic drops were prescribed in the emergency department for pain control, after which patients developed persistent ocular pain, epithelial toxicity with impaired healing, and significant loss of vision. On consultation with the ophthalmology service, each patient was instructed to discontinue the topical anesthetic drops but to continue topical antibiotics. Soon thereafter, symptoms resolved, and the corneas healed within 72 hours. This report emphasizes the harm that can result from injudicious and/or unmonitored use of topical anesthetics for corneal abrasions. We advise emergency medicine and primary care providers against providing topical anesthetics to patients with corneal abrasions.

## Introduction

Corneal abrasions, disruptions of the epithelial surface of the cornea, are usually classified as spontaneous (due to underlying corneal dystrophy, for example) or traumatic (due to flash burns, foreign bodies, contact lens use, or other causes) [1,2]. They constitute a significant proportion of ocular injuries and are responsible for 4% of all emergency department visits (EDs) in the United States and Europe. The most commonly affected patients are young working males [2-5].

Presenting symptoms include conjunctival injection, blurred vision, acute pain, tearing, photophobia, and foreign body sensation [3,4]. Early detection and treatment are critical because persistent abrasions may lead to significant vision loss, infectious keratitis, traumatic iritis, intractable pain, corneal scarring, and corneal perforation [1,6,7]. Pressure patches and mydriatics have long been used to treat corneal abrasions, but randomized controlled trials (RCTs) show their limited ability to decrease pain in most patients [8-12]. Additionally, patches and bandage contact lenses have been associated with corneal hypoxia, which increases the risk of infection [12,13]. As a result, corneal abrasions are more frequently treated with topical ophthalmic antibiotics and nonsteroidal anti-inflammatory drugs (NSAIDs) [12]. 

Data supporting the efficacy of topical NSAIDs remains inconclusive despite decades of use [14]. Administration of topical antibiotic ointments and NSAIDs has been shown to prevent post-traumatic corneal ulcerations in a two-year prospective cohort study and meta-analysis of five RCTs, respectively [15,16]. However, a meta-analysis of nine RCTs found no compelling evidence supporting the use of NSAIDs in traumatic corneal abrasions [17], and the use of NSAIDs should be carefully considered in all patients because of their high costs and increased incidence of adverse effects on the cornea [18].

Topical anesthetic use in patients with corneal injuries is controversial, but emergency medicine providers frequently dispense them [19]. Recent (February 2024) consensus guidelines from the American College of Emergency Medicine Physicians (ACEP) state that self-administered commercial ophthalmic anesthetic use for simple corneal abrasions is safe when given every 30 minutes during the first 24 hours after presentation [20]. Topical anesthetic use is appealing because patients initially tolerate drops well, and corneal anesthesia is easily and rapidly achieved; however, their unsupervised use may result in patient abuse and toxicity to the cornea [21,22].

Our report documents three cases in which the healing of small corneal abrasions was delayed or worsened due to short-term, self-administered use of topical anesthetic drops. In each case, subspecialty care evaluation and intervention were required to correct the iatrogenic injury.

## Case presentation

Case one

**Figure 1:**
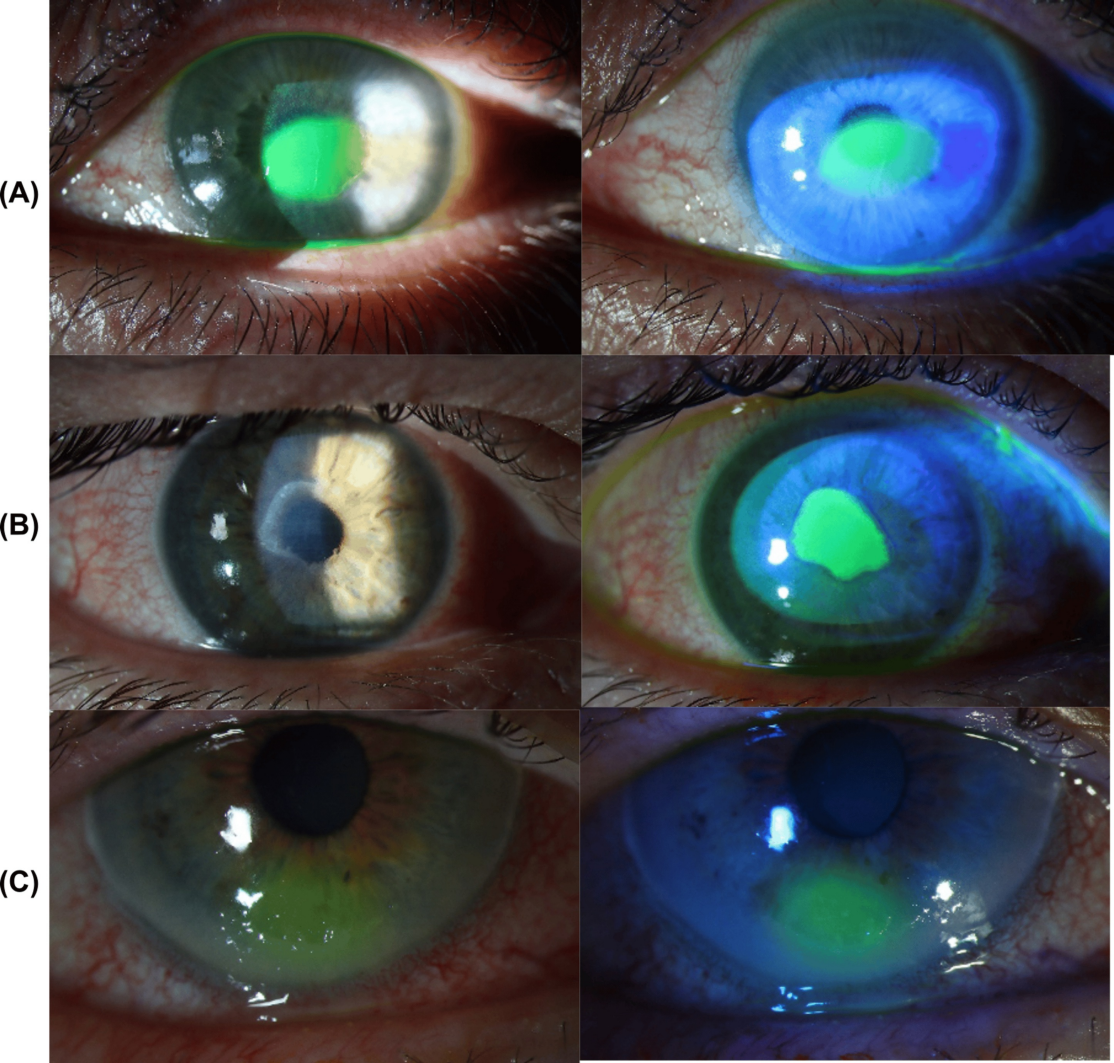
Slit lamp photos highlighted with normal light (left) and cobalt blue light (right) following fluorescein staining (A) Patient one: central corneal abrasion in the left eye; (B) Patient two: central corneal abrasion in the right eye; (C) Patient three: inferior corneal ulcer in the right eye.

A 59-year-old male underwent a cardiac procedure under general anesthesia and awoke with debilitating pain in the left eye (OS). He was prescribed a topical antibiotic (ofloxacin) and topical anesthetic drops (proparacaine hydrochloride ophthalmic solution, 0.5%) by an emergency medicine provider. One day later, on examination in the ophthalmology clinic, the patient was found to have bilateral keratitis, a conjunctival injury, and a central corneal abrasion (measuring 2.5 mm x 3.5 mm) without a foreign body (Figure 1A). Best corrected visual acuity (BCVA) measured 20/50 in the right eye (OD) and 20/60 OS. A non-healing corneal abrasion was diagnosed. A bandage contact lens (BCL) was placed, and the patient was instructed to discontinue the use of topical anesthetic drops, continue antibiotic drops, and begin artificial tears in both eyes (OU). Three days later, the superficial keratitis resolved OD, and the corneal abrasion healed OS. Best corrected visual acuity had improved to 20/20 OU. 

Case two

A 42-year-old male underwent colorectal surgery under general anesthesia and noted pain, blurred vision, and light sensitivity OD after awakening. A topical anesthetic (proparacaine hydrochloride ophthalmic solution, 0.5%) was prescribed by an emergency medicine provider. Five days later, the patient was seen in the ophthalmology clinic for worsening pain due to a central corneal abrasion OD (measuring 3 mm x 3 mm) without a foreign body (Figure 1B). Best corrected visual acuity measured 20/100 OD and a non-healing corneal epithelial defect was identified. The patient was instructed to discontinue anesthetic drops, was started on polymyxin B/trimethoprim ophthalmic solution (Polytrim) four times daily, and a BCL was placed. Three days later, BCVA improved to 20/20 OD, the pain had resolved, and the corneal abrasion had healed completely. 

Case three

An 81-year-old female with a history of narrow angles, cataracts, and posterior vitreous detachments OU presented to the ED because of significant eye pain. She was diagnosed with a corneal abrasion OD and was prescribed a topical antibiotic (moxifloxacin) and anesthetic (proparacaine hydrochloride ophthalmic solution, 0.5%) to use twice daily. Four days later, the patient was evaluated in the ophthalmology clinic for a corneal ulceration OD. An epithelial defect in the inferior peripheral cornea with associated stromal thinning consistent with medicamentosa was seen OD (Figure 1C). A BCL was placed, and the patient was instructed to stop the proparacaine drops while continuing the antibiotic drops. Three days later, the patient reported significantly improved comfort OD, and the corneal abrasion had resolved. Visual acuity returned to her baseline of 20/100. 

## Discussion

We report three cases in which anesthetic drops delayed epithelial healing in patients with corneal abrasions. Within 72 hours of stopping the anesthetic drops, all corneal abrasions had resolved, and visual acuity recovered. Based on our evaluation of the literature and our experience with these three cases, we strongly discourage the unsupervised use of topical anesthetics to treat corneal abrasions. 

Support for topical anesthetic use comes from two prospective, double-masked trials in which closely monitored self-administration of a topical anesthetic (tetracaine hydrochloride ophthalmic solution, 1%) over 24 to 72 hours was found to effectively relieve pain without worsening vision or delaying wound healing in patients after photorefractive keratectomy (PRK) [23,24]. A randomized controlled trial (RCT) found that the use of topical anesthetics in patients with uncomplicated corneal injuries did not result in any adverse effects over a two-week period. However, the trial’s low retention rate at 36-to-48 hours (34%) and the short duration of anesthetic use limited its ability to determine whether anesthetic use delayed corneal re-epithelization [25]. Two retrospective studies investigated the effects of 24-hour treatment with topical anesthetics in 116 and 1576 ED patients with corneal abrasions [26,27], and both concluded that topical anesthetics were safe, though several adverse effects were reported in a "non-simple corneal abrasion group" [26,27]. Importantly, the topical anesthetic use was controlled and did not extend past 48 hours. The literature, however, suggests that long-term, uncontrolled use of topical anesthetics causes secondary infections, cytotoxic effects, and delayed wound healing [25,28].

Topical anesthetics are frequently prescribed by emergency medicine providers but unsupervised outpatient use is strongly discouraged by corneal specialists due to the high likelihood of patient abuse and corneal toxicity [29-34]. The cornea is the most densely innervated structure within the body, and injuries produce excruciating pain [19,35]. Pain signals from the injured cornea stimulate the trigeminal nerve to improve lubrication by increasing both the blink reflex and tear production. This bathes the cornea with growth factors such as insulin-like growth factor 1, substance P, glial cell-derived neurotrophic factor, nerve growth factor, and neurotrophin 3, which nourish the epithelium and promote healing [19,36,37]. The pain pathway is essential for corneal healing, and the absence of corneal sensation that occurs with frequent topical anesthetic use downregulates growth factor synthesis and leads to epithelial loss, abnormal neuronal regrowth, development of neuropathic pain, and fibrosis. This can occur when patients have unlimited access to topical anesthetics [19,37-39].

Topical anesthetics such as proparacaine and tetracaine produce effective anesthesia of a brief duration (30 minutes), so maintaining anesthesia requires frequent, repeated administration. Anesthetic keratopathy due to topical anesthetics has been documented after 10 days to six months of use [29-34], though our patients developed anesthetic keratopathy after much shorter periods (one day, five days, and four days, respectively). This highlights the dangers that can result from even a single bottle of anesthetic that is used within the 24-hour timeframe as recommended by the ACEP consensus guidelines [20]. 

Studies supporting the use of topical anesthetics to treat corneal injuries are limited by small sample sizes [23-25] and retrospective designs [26,27]. Three prospective, double-blind trials investigated the effects of topical anesthetics in patients following PRK surgery [23,24,40]. Though each reported positive outcomes with anesthetic use, their results are not generalizable to corneal abrasions since the effects of anesthetics on sterile, surgically induced PRK wounds cannot be directly compared to the nonsterile corneal abrasions seen in our report. The administration of dilute (0.05%) topical proparacaine to 34 patients did not limit epithelial healing, but dilute proparacaine is not readily available in clinical settings and must be prepared by compounding pharmacies [40]. Importantly, these patients were treated under aseptic conditions and were closely monitored by an experienced ophthalmologist to assess corneal healing.

When seeing patients with painful corneal abrasions, emergency physicians must consider several factors, including pain control. Prescribing topical anesthetics to these patients constitutes an effective short-term pain control strategy, but it risks longer-term epithelial dysfunction with prolongation of pain and the development of corneal scarring. Alarmingly, this practice pattern is employed by emergency medicine providers, and its use has increased over time [19].

Within the ophthalmic community, anesthetic use in patients with corneal abrasions is discouraged. After discharge from the ED, patients with corneal healing problems are often seen by ophthalmologists and not ED physicians. In a recent (May 2024) editorial, Chuck et al. discussed the challenges faced by the fragmented care process, emphasizing discrepancies in the treatment and evaluation of corneal abrasions by two distinct medical specialties [41]. In light of the various shortcomings of previous evidence and an unmet burden of proof for providing topical anesthetics to patients with corneal abrasions, as determined by a rigorous systematic analysis [42], the authors strongly contested the new ACEP consensus guidelines. To allow for data-driven guidance, they advocated a robust approach that emphasizes tightly controlled trials, exams with ophthalmologists, and long-term follow-up [41]. An anonymous survey to 75 international corneal specialists found that most respondents disagreed with discharging corneal abrasion patients with topical anesthetics (100% disagreed) or discharging them with a 24-hour supply (89% disagreed) [19]. Considering these findings, we believe that the inherent risks of topical anesthetic use for corneal abrasions outweigh any possible benefits, and drops should not be prescribed for outpatient use.

## Conclusions

Self-medication with topical anesthetic drops was found to significantly delay wound healing in three patients with corneal abrasions. To our knowledge, this is the first report of patients developing anesthetic keratopathy from unmonitored anesthetic use within a rapid time frame of one to five days. This emphasizes the serious risks associated with using a single bottle of anesthetic within the 24-hour period recommended by the ACEP consensus guidelines. In conclusion, the practice of routinely prescribing topical ophthalmic anesthetics by emergency medicine providers to patients with corneal abrasions should be strongly discouraged, with topical antibiotics and lubricating drops serving as preferred alternatives for treating corneal abrasions.
